# One-house one-person testing: Strategical plan to limit COVID-19 spread in stage three in the developing world

**DOI:** 10.1017/ice.2020.200

**Published:** 2020-05-06

**Authors:** Sheikh Muhammad Ebad Ali

**Affiliations:** Dr Ruth KM Pfau Civil Hospital Karachi, Saddar, Karachi, Pakistan

*To the Editor*—Coronavirus disease 2019 (COVID-19) is a respiratory viral disease discovered in Wuhan Province of China in November 2019, but soon the SARS-CoV-2 virus has spread across the entire world, and the World Health Organization (WHO) declared COVID-19 a pandemic in March 2020. In the hour of crisis, the only preventive measures were strict quarantine; hygiene maintenance with regular handwashing; covering mouth, nose, eyes, and ears; risk screening at airports and railways^1^; and social distancing. A strategy was proposed after the advent of a virus-specific molecular polymerase chain reaction (PCR) test called “mass screening” that involved testing nasal and throat swabs from the random population to assess viral spread and to isolate those infected from the healthy population.^2^ The strategy gained widespread approval in developed countries, and huge random populations were screened. However, the cost of testing was an obstacle for developing nations; governments intended to adopt this strategy but could not due to the overwhelming expense.

Consequently, COVID-19 outbreaks across the globe have continued to occur, with less PCR testing of the population because of overburdened healthcare system and economic limitations under strict lockdowns. As of April 23, 2020, 2,630,516 confirmed COVID-19 cases, along with 183,924 COVID-19 deaths and 58,139 COVID-19 patients in critical condition have been reported.^3^ Most of the cases reported have been attributed to local transmission through respiratory droplets. Most countries have implemented strict lockdowns, but the results have not been satisfactory in terms of local transmission control and adverse economic effects.

I propose a method of screening that can be used in remote areas and developing nations during stage 3 of the COVID-19 pandemic; it is cost efficient and has a high probability of isolating asymptomatic cases. Theoretically, the strategy could also ease the lockdowns rapidly and thus help mitigate the global economic crisis during stage 3 of this pandemic. This technique may be highly useful in overcrowded and slum areas that show higher levels of local transmission from asymptomatic cases.

As reported in previous studies, COVID-19 spreads rapidly through droplets, and the probability of infection is increased if a person comes in contact with any infected patient. However, asymptomatic cases, which may be as high as 84% of all cases, may have similar transmission risks as symptomatic patients. For those quarantined in their homes, the most vulnerable victims can be close family members of infected patients living in the same house, which is termed a family cluster (Table 1).

**Table 1.** Studies Reporting Family Clusters

Study	Familial Cases, No.	Index Patient, No.
Jiang XL, et al^4^	6	2
Ye F, et al^5^	2	3
Zhang J, et al^6^	4	1
Qian G, et al^7^	7	2
Li P, et al^8^	4	1
Guan Q, et al^9^	6	1

**Fig. 1.**
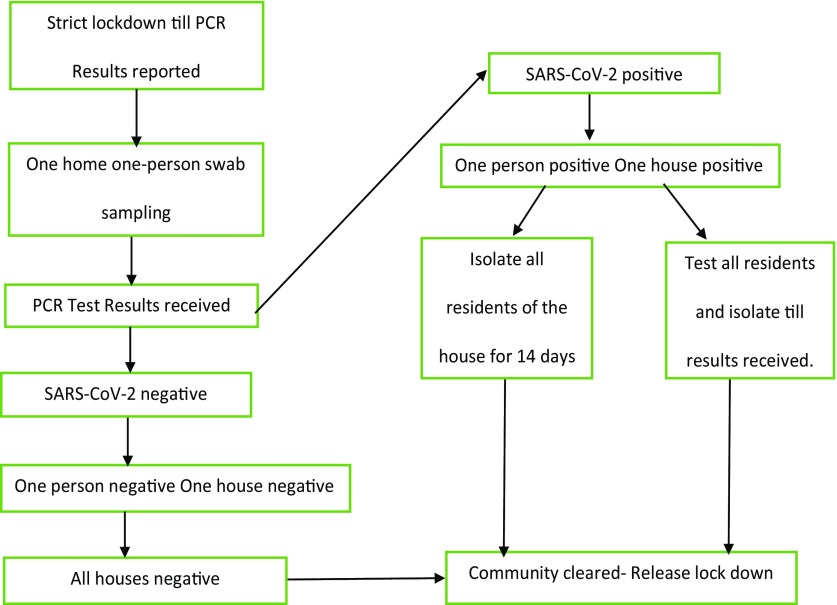
One-house, one-person plan.

In the strategy I propose, instead of random sampling, areas under lockdown should be allocated within boundaries, and a single person from each household should be tested using swabs collected door to door. The PCR results are available within 48 hours. If a person from 1 house is positive, the whole family should be either screened or isolated for the following 14 days. If the person is negative, then the whole family can be considered negative (Fig. 1).

For example, India, with a population of 1.353 billion, cannot test each citizen, and there is a high probability of missing COVID-19 cases in random sampling, which might worsen the situation once lockdown is lifted. However, India has some of the largest slum areas in the world, where 10–20 people reside per 27.9 m^2^ (~300 square feet), producing a very high risk of spread. Hence, if 1 person from the slum gets tested, there is a high probability that the test results reflect the status of those nearby. Rather than testing the whole slum, 1 person from every hut in each slum could be tested. This approach would reduce the number of tests and help to end the lockdowns within 4–5 days.

Although this strategy is not comparable with mass screening, it may offer a substantial decrease in the burden of disease, especially in countries with larger populations and limited resources. I request that researchers conduct cross-sectional studies to execute this plan, which could save lives by preventing local transmission from asymptomatic COVID-19 cases.
